# On Reliable and Efficient Data Gathering Based Routing in Underwater Wireless Sensor Networks

**DOI:** 10.3390/s16091391

**Published:** 2016-08-30

**Authors:** Tayyaba Liaqat, Mariam Akbar, Nadeem Javaid, Umar Qasim, Zahoor Ali Khan, Qaisar Javaid, Turki Ali Alghamdi, Iftikhar Azim Niaz

**Affiliations:** 1 Institute of Space Technology, Islamabad 44000, Pakistan; tayyaba.bet091@gmail.com; 2 COMSATS Institute of Information Technology, Park Road, Islamabad 44000, Pakistan; mariam.akbar@gmail.com (M.A.); ianiaz@comsats.edu.pk (I.A.N.); 3 Cameron Library, University of Alberta, Edmonton, AB T6G 2J8, Canada; umar.qasim@ualberta.ca; 4 Internetworking Program, FE, Dalhousie University, Halifax, NS B3J 4R2, Canada; zahoor.khan@dal.ca; 5 International Islamic University, Islamabad 44000, Pakistan; qaisar@iiu.edu.pk; 6 College of CIS, Umm AlQura University, Makkah 11692, Saudi Arabia; ta_ghamdi@hotmail.com

**Keywords:** underwater wireless sensor networks, routing protocol, cooperative communication, diversity, reliability, throughput efficiency, energy optimization, delay optimization, mobile sink, autonomous underwater vehicle

## Abstract

This paper presents cooperative routing scheme to improve data reliability. The proposed protocol achieves its objective, however, at the cost of surplus energy consumption. Thus sink mobility is introduced to minimize the energy consumption cost of nodes as it directly collects data from the network nodes at minimized communication distance. We also present delay and energy optimized versions of our proposed RE-AEDG to further enhance its performance. Simulation results prove the effectiveness of our proposed RE-AEDG in terms of the selected performance matrics.

## 1. Introduction

Underwater WSNs are getting their deserved economical, social and ecological importance due to its extended range of applications such as seismic monitoring, data logging, military applications, disaster management, etc. For UWSNs multiple unmanned underwater vehicles are deployed to form a network. Deployed sensors form a collaboration and help humanity in underwater resources’ exploration. Generally these nodes are equipped with the required sensors and are responsible for collection of scientific or environmental information from the defined atmosphere.

Under water is a harsh environment and poses a lot of unique challenges [1,2] like higher latency, greater attenuation levels, higher transmission energy, lower data authenticity, difficult extraction of data, unexpected failure of node, limited availability of bandwidth, confined battery power, etc. Therefore, to communicate data in underwater environment radio signals require larger antennas and demand higher transmission power which is purely unfeasible. Aforementioned are the few reasons acoustic channels are considered to be more appropriate for UWSN communications as compare to radio waves [3]. For an underwater environment they are proved to be immune towards attenuations, signal distortion, end to end delays, higher BER due to multi path fading, etc.

A number of protocols have been designed by the scientists to affix the above stated issues. Whereas; the key point to insight here is that, un till now most of the designed protocols works on the network’s energy conservation while does not contributes towards higher level data reliability. Keeping all that challenges in mind a protocol was required that should be well organized, cost effectual and handles energy to upgrade the data validity factor for enhanced outcomes. Therefore, as a consequence of these mentioned features we propose a routing protocol named as RE-AEDG. It is focused on magnifying the system reliability by improvement in throughput rate and successful packets delivery ratio at the surface sink. Moreover, in order to attain the stated objectives, a strategy known as cooperative routing is applied in RE-AEDG. This scheme is appropriate because it substantially boosts the spectrum utilization without placing any extra cost liability over the system.

In cooperative routing, each in range node plays the role of a potential relay and eventually the destination receiver hears the packets from multiple transmitters. It combine them using some predefined diversity combining techniques e.g., Maximal Ratio Combining technique (MRC), Equal Ratio Combining technique (ERC) and others [4,5]. However, these all advantages comes at the cost of faster battery power dissipation mechanism that leads towards an ill-favored state known as energy-hole problem.

Mobile sink appears to alleviate this problem through network load balancing. Furthermore, it cuts short the communication paths through eliminating source and destination connection. Although the mobile sinks are highly desirable due to lots of advantages they pose in terms of network lifetime and throughput, their mobility mechanism causes a-lot of challenges in system designing process, moreover, complicates the efficient distributed data routing. Controlled sink mobility restricts the path selection frequency hence, reduces the protocol overhead caused by sink mobility.

**Figure 1:**
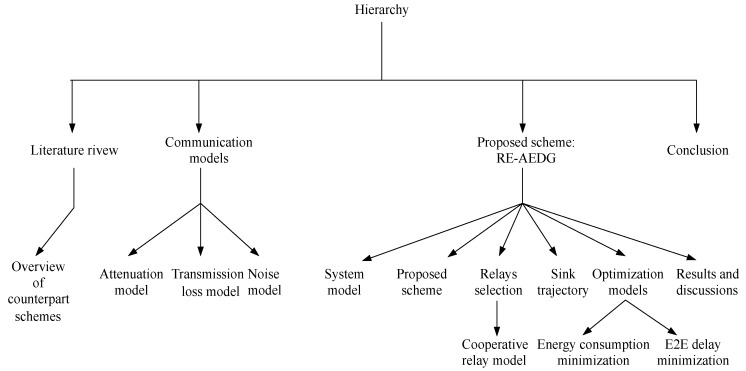
Paper hierarchy.

The rest of the paper is organized as follows. Section 2 describes related work; overview of counterpart schemes is given in Section 3; Section 4 presents underwater acoustic communication models; Section 5 presents system model; Section 6 discusses the proposed scheme; Section 7 deals with possible relay selection; Section 8 describes AUV trajectory and Section 9 defines the optimization models for the proposed scheme; Section 10 elaborates the applications of the proposed model; In Section 11, performance efficiency of the proposed scheme is validated via simulations. The hierarchy of paper is also presented in Figure 1.

## 2. Related Work

In source node based retransmission techniques, acknowledgement from the receiver node stops further re transmissions of the same packet. However, the source node is subjected to several retransmissions which makes network unstable. In [6], authors improve the ARQ protocol such that the receiver node asks for retransmission of the same packet from the cooperative nodes. Thus, it leads to network stability which in turn leads to network lifetime prolongation.

In [7] Dynamic node cooperation in UWSNs is presented for UWSNs. This mechanism never asks retransmissions from the same node whose data was not-decoded formerly. The receiver asks for a retransmission from the node that has overheard the same packet and got better channel conditions. In this protocol the relay nodes are preferred on the basis of link quality and SNR.

Similarly [8] uses channel statistical information to select the best relay. It takes into account both the incurred propagation delay and transmission time. The authors also formulated an optimum one way packet transmission (OPT) time. This protocol is biased towards source relay channel as compare to relay destination channel.

In [9], authors present a unique cooperating nodes selection norm which is based on propagation delay. This scheme considerably minimizes the amount of required retransmissions among the nodes. Thus, the selection of cooperating node may leads to efficacious energy consumption of the network.

Cooperative communication is an energy expensive technique due to retransmissions of same data packet. In [10] sink mobility is introduced to minimize the energy consumption cost of the network. AUV follows an elliptical trajectory and takes sojourn stops after regular intervals. In this way, the communication distance between the nodes and surface sink is minimized which leads to network lifetime prolongation.

In [11] EASR protocol has been proposed. It performs sink reallocation for network lifetime prolongation. Sink is triggered for a next shift in its position depending upon the residual energy of some specific region inside the network. Maximum Capacity Path (MCP) is a dynamic routing method that has been adopted to report data from source to sink. Moreover nodes are also considered to be capable of energy aware transmission range control program.

${\left(ACH\right)}^{2}$ a routing scheme for maximizing throughput and network lifetime has been proposed in [12]. It is cluster based scheme whose uniqueness is uniform distributed load over the cluster heads. Cluster head selection is performed in two separate phases; first is the random nodes’ selection, followed by the filtering of the candidate nodes on the basis of their residual energies; Secondly, for the sake of well distributed cluster heads their number is optimized by determining their intermediate distances. Moreover nodes receiving better signal strength from central base station communicate directly to it, hence, shorter transmission paths.

Co-UWSN proposed in [13] is a routing protocol that combats fading in data transmission process using cooperative communication among nodes. Relay nodes are selected on the basis of the signal strength and distance information. Therefore, significant decrease in path loss consequently, better throughput and enhanced data integrity.

Jiabao Cao and few others have proposed a balance transmission mechanism in [14]. It divides the data routing process into two segments. In the first step, it creates a tree of nodes which is actually a path, a packet follows in data transmission process. This path is created on the basis of optimum transmission distance. On the second stage data routing algorithm is designed. Multihop and single hop both methods of communication are employed to balance the energy consumption. Energy gradation concept is also used in order to decide the transmission mode of nodes. Simulations and results prove the reliability of the protocol in the enhancement of network lifetime.

A hybrid approach consisting of dynamic and static clustering has been employed in [15]. This technique was developed to overcome the energy holes creation in the network. Authors divide the network into fixed logical regions to avoid the unbalanced energy consumption. Furthermore cluster head selection is done via dynamic and static selection procedures.

In [16] authors took multiple pre-existing protocols and proposed their improved versions in terms of delay sensitivity. In order to minimize the end to end delay all the schemes formulates delay-efficient priority factors moreover, enhances the holding time to delay sensitive holding time.

While considering the density and node’s location a load balancing clustering algorithm has been proposed in [17]. Cluster radius is adaptive in the proposed protocol and adjusts on the basis of nodes density in certain region. Higher the nodes density, lower would be the cluster radius; which has been formulated based on density and nodes’ distance with base station. Protocol has been proved to be more stable and enhances the network lifetime as well.

In [18], authors consider the node’s degree residing in the network. Node’s degree is considered as the number of neighbors residing in a certain radius. This node’s degree is employed in computation of preferable clustering and relay nodes. The aforementioned protocol is proved to be an energy efficient protocol because higher degree nodes are elected as cluster heads and relays nodes. Therefore, larger number of nodes is accommodated by lesser cluster heads.

In [19], an optimization approach EDIT has been proposed that optimizes the two delicate network performance metrics, energy consumption and end to end delay. Two different types of distances between cluster head and member nodes have been considered that defines unique tradeoffs in between energy consumption and delay.

Another clustering protocol has been proposed in [20]. In this proposed protocol cluster head is selected through formulating residual energy and the number of neighbors surrounding a node. Moreover, a shortest path algorithm has been employed to obtain a route optimization technique for data forwarding phase. Hence, an energy efficient clustering strategy has been proposed to elongate the network lifetime.

In [21] a clustering protocol has been suggested. It performs by distributing the network into fan shaped smaller cells. Furthermore, different energy saving methods have been proposed considering the above mention network layout. Whereas, in [22] LAFR technique has been proposed. In this technique network energy consumption has been cut short by limiting the number of links connected to the upstream node. To prevent data flooding authors employed the routing table being maintained by the downstream nodes. LAFR considers the beam width and three dimensional data transmission by sensors.

iAMCTD is a protocol, that has been proposed in [23]. Authors sub divided the acoustic region into three layers. Furthermore, each layer has been assigned with a unique forwarding function. Each forwarding function selects the forwarder node by optimally minimizing the environmental constraints dominant for that specific network layer.

In [24], AURP has been proposed that employs multiple AUVs as data relaying nodes. AUVs cut short the transmission distance and aids in efficient data gathering process. AUVs follow a dynamically decided fashion to achieve the maximal network performance in terms of data delivery ratio and energy consumption.

SPARCO is a cooperative based routing protocol suggested in [25]. The proposed protocol is a hybrid transmission technique whereas, best relay selection mechanism depends upon the instantaneous path information and distance among neighboring nodes. The protocol has been proposed to lower path loss and improve network stability.

Cylindrical networks have been considered in [26] and chain based routing has been employed. This paper proposed multiple schemes elaborating the routing mechanism using 1 to 4 inter-connected chains of sensor nodes. These chains are formed through calculation of optimum paths both locally and globally. A four chain based scheme presents a significant performance in terms of load balancing and energy consumption.

LESCA is another protocol that has been proposed in [27]. Under consideration network is clustered using K-ways technique whereas, average energy, distance to BS, and distance to clusters centers are the few parameters that are used in cluster heads determination process. LESCA reduces energy consumption and lifetime gains of the network.

WDFAD-DBR is a protocol presented in [28] that implements a technique to avoid the coverage holes creation in a network. Depth difference among nodes is considered as the key factor while selection of forwarding node for data transmission. Furthermore, nodes’ forwarding area and nodes’ eligible neighbors are also confined to reduce the energy consumption. Whereas, in [29], coverage hole problem has also been addressed in a unique way which is based on minimum critical threshold constraint. BLW-MCT has better performance in network scalability and outperforms counterpart schemes in terms of coverage holes.

In [30], a protocol has been presented that adaptively varies the transmission range of nodes hop by hop depending on neighbors’ density. Moreover, holding time is also optimized to overcome the end to end delay constraint among network. Above mentioned protocol is proved to perform well if implemented among sparse acoustic networks.

Balanced clusters is the core issue been discussed in [31]. A fitness function has been formulated to reduce the overall energy consumption of the network. GAECH is proved to produce better results as compare to the counterpart schemes in terms of network lifetime and stability period.

For random deployment scenarios heterogeneous communication ranges could produce better results in terms of network connectivity rate. This is the key motivation being adopted in [32]. In this protocol heterogeneous transmission range is also fused with the concept of aggregate contribution degree.

Another protocol has been proposed in [33] that works by dividing the clusters into further smaller clusters. A path-node is selected for data gathering purpose at every sub cluster. Afterwards AUVs take a data gathering tour to visit path-nodes for data gathering. Relay nodes are circulated on the bases of residual energies and are selected out of the member nodes by the cluster head.

In [34] GPNC has been suggested that exploits the greedily data forwarding approach. It effectively reduces retransmissions by employing partial network coding moreover, achieves higher packet delivery ratio with efficient energy consumption. Whereas, another routing technique has been proposed in [35] that forwards the packet on a link by carefully observing the link expiration time. Protocol provides reliability to the system through the exchange of acknowledgement messages. Nodes search for alternative link if the link expiration time exceeds packet’s delivery time plus the time being consumed in exchange of acknowledgement messages. This protocol achieves better end to end delays in contrast to counterpart schemes.

As discussed earlier, AUVs contribute to performance improvements in many ways, however they create new problems too. In [36] a protocol is proposed that is named as LVRP. AUVs causes the rapid changes in network topology which results in protocol overhear. LVRP formulates a mechanism that is benefited by the usage of AUVs besides that minimizes the packet overhead by the employment of a controlled mechanism that reduces the requirement of rapid control packet exchange among nodes.

In addition to that there is another protocol called AEDG [37] that also contributes in protocol overhead suppression generated by the movements of AUVs. It associates a set of static nodes with AUVs which are responsible to gather data from surrounding nodes and to track the AUV’s current sojourn stop. This technique appears to be an energy conservation method for UWSNs. A brief overview of existing schemes is presented in Table 1.

## 3. Overview of Counterpart Schemes

AEDG and LVRP are chosen for comparison because of their conceptual relevancy with RE-AEDG. In LVRP a model is designed which considers multiple mobile sinks following unconstrained sink trajectories. In the data transmission process control packets are generated in order to track the recent sojourn stop of sink. In LVRP, the major objective is to minimize the packet overhead, caused due to the flow of these control packets. Therefore, at LVRP the packet overload is reduced through proper linking of an anchor node to the sink. Anchor node gathers the data on the behalf of sink and the sink issues new routing updates only when it switches its sojourn stop and no more remains in its transmission range. It is selected through proper election from the nearest member nodes, moreover; it is a casual node and is responsible for making a connection between nodes and sink.

**Figure 2:**
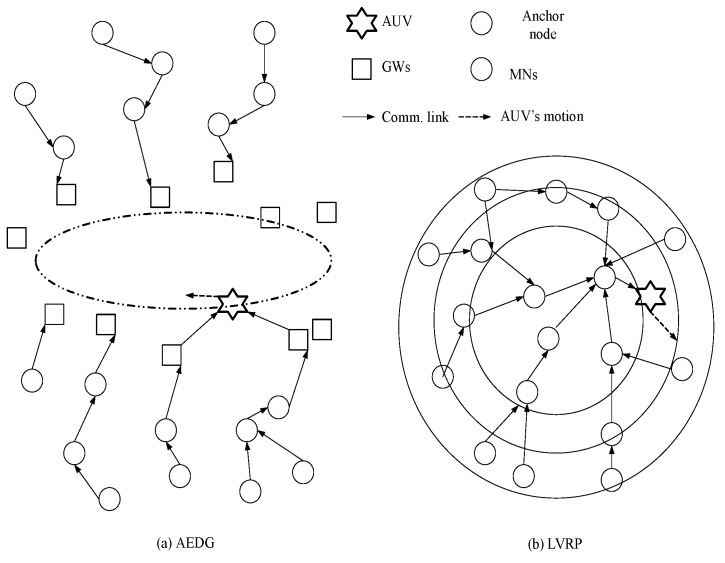
Network architecture.

Whereas in AEDG, heterogeneous nodes are deployed in vicinity of a predefined elliptical AUV trajectory, nearer nodes are GWs and the farther ones are MNs. GWs gather data from MNs and hold it until AUV approaches them. Deployment of GWs reduces the control packet overhead as lesser number of nodes track the recent AUV locality. MNs form a chain among themselves with a GW node as chain head. Moreover, number of nodes connected in a chain is also restricted to avoid longer intercommunication distances. Conservation of energy is the distinguishing factor of AEDG. Thus the design objective of our proposed technique is the effective utilization of that conserved energy to intensify network’s operational efficiency in terms of network lifetime and throughput rate. Pictorial view of AEDG and LVRP with single AUV is shown in Figure 2.

## 4. UWSNs: Communication Models

As discussed in the introduction section, the characteristics of acoustic wave propagation are different from the characteristics of radio wave propagation. Thus, precise models are needed.

### 4.1. Attenuation Model

Underwater acoustic signals experience power level degradation. Attenuation is calculated for distance “*R*” in meters and frequency “*f*” in KHz and is given as follows:

(1) $$A\left(R,f\right)=R^{k}\;a_{u}{\left(f\right)}^{R}$$

$a_{u}\left(f\right)$ is measured in dB/km where as k differs with geometry of propagation. It is 2 and 1 for spherical and cylindrical spreading respectively [38,39,40]. The absorption coefficient *a* is calculated using Thorp’s empirical formula:

(2) $$10loga\left(f\right)=0.11\frac{f^{2}}{1+f^{2}}+22\frac{f^{2}}{4100+f^{2}}+2.75\;\times \;10^{-4}f^{2}+0.003$$

### 4.2. Transmission Loss Model: Deep and Shallow Water

Transmission loss also known as propagation loss, defines the decrease in energy level of signal as wave propagates through the specified medium. For shallow water, the propagating acoustics signals experience cylindrical spreading [41,42].

For shallow water transmission loss is calculated as follows:

(3) $$TL=10log\left(R\right)+a_{u}\left(R\right)\;\times \;10^{-3}$$

For deep sea transmission loss is calculated as follows:

(4) $$TL=20log\left(R\right)+a_{u}\left(R\right)\;\times \;10^{-3}$$

### 4.3. Noise Model

Underwater noise is due to three main factors: scattering, absorption and spreading loss. These losses are mainly caused by unsteady movement of water, ships motion for the sake of tourism or trade, military operations, etc.

Total noise power spectral density is calculated in the following set of equations:

(5) $$NL\left(f\right)=N_{t}\left(f\right)\;+\;N_{s}\left(f\right)\;+\;N_{\omega }\left(f\right)\;+\;N_{th}\left(f\right)$$

whereas;

(6) $$10logN_{s}\left(f\right)\;=\;40\;+\;20\left(s\;-\;0.5\right)\;+\;26log\left(f\right)\;-60log\left(f\;+\;0.03\right)$$

(7) $$10logN_{\omega }\left(f\right)\;=\;50\;+\;7.5\;\omega^{1/2}\;+\;20log\left(f\right)\;-\;40log\left(f\;+\;0.4\right)$$

(8) $$10logN_{th}\left(f\right)\;=\;-\;15+\;20log\left(f\right)$$

All the four noise factors directly depend on frequency, that’s why fluctuation in frequency effects the communication undergoing the environment.

## 5. System Model

**Figure 3:**
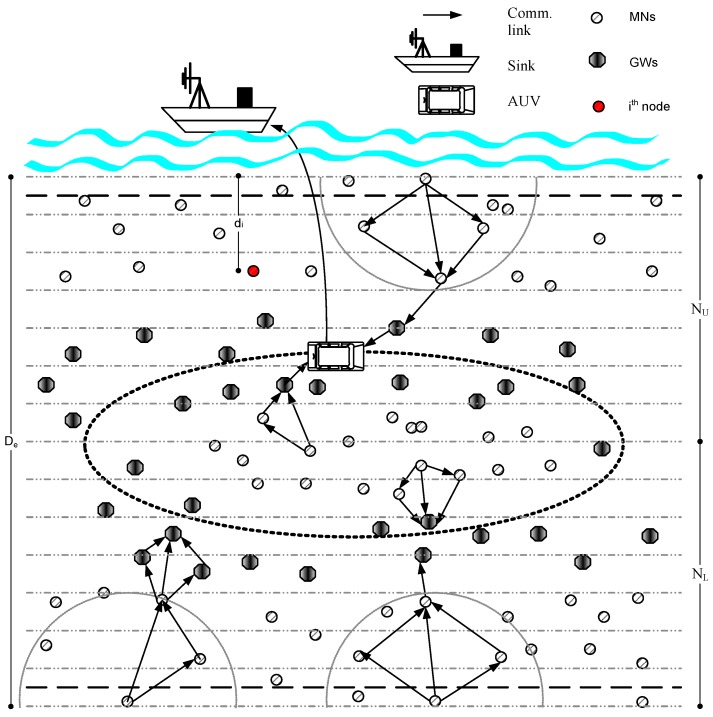
Network architecture.

Figure 3 shows that nodes are randomly deployed in the network field. Depending upon the features that are represented in Table 2, we categorize the network nodes into member nodes (MNs), gateway nodes (GNs) and autonomous underwater vehicle (AUV). In this work, the following assumptions are made:

- MNs continuously sense the required information from the surroundings.
- Nodes repeatedly measure their residual energies and deliver their energy states towards their predecessors using a control packet.
- Upon reception of location information from the in-range nodes, each member node maintains and updates its neighbors list.
- Nodes are capable to accept or discard a packet on the basis of BER. To enhance the data reliability they may also ask forsingle or double retransmissions of the erroneous data packet from the sender node.

Furthermore, we use three different transmission ranges that correspond to different regions called slices. Nodes that belong to the same slice are the *Sibling nodes*.

**Rule** **1:**  *Sibling nodes are restricted to do not exchange data packets with each other.*

This rule minimizes the number of hops between source and destination nodes. However, eligible neighbors are also minimized in number. This situation is highly undesirable during later simulation time when most of the network nodes are dead. To avoid this situation, an intermediate rule is defined, which provides the solution by dividing the 1st slice further into two layers. Layering is conducted by partitioning the width of slice 1 into two regions, such that upper and lower region of 1st slice makes the 1st and 2nd layer respectively. Source node itself lies in the 2nd layer.

## 6. RE-AEDG: The Proposed Scheme

With the help of heterogeneous nodes’ deployment and a central AUV, AEDG proved its improved network performance in terms of network energy consumption and E2E delay. However, factor of reliability has not been considered much while network designing. Therefore, in this paper we propose RE-AEDG which is the improved version of AEDG in terms of data reliability. RE-AEDG takes the advantage of that conserved energy and better utilizes it for secured data transmission at the destination node.

Underwater networks are generally harsh environments consisting of larger area networks. While considering the limited energy of nodes, data transmission in this sort of network could be challenging. Therefore, underneath is the proposed scheme for RE-AEDG that shows how nodes are deployed and grouped together to create network layers for relatively convenient data collection and transmission process. Let *N* denotes the set of sensor nodes; N=n whereas, deployed network nodes are fairly portioned into $N_{U}\;\;and\;\;N_{L}$ . For further elaboration consider a deployed node *i* that may belong to $N_{U}$ or $N_{L}$ depending upon the following conditions:

(9) $$i\;\epsilon \left\{\begin{array}{c} N_{U}\;if\;d\left(i\right)\leq \frac{1}{2}\left(D_{e}\right)\\ N_{L}\;if\;d\left(i\right)>\frac{1}{2}\left(D_{e}\right) \end{array}\right.$$

where, d(i) be the depth of considered node and D(e) be the depth of the network under examination. Furthermore, the above written equation states that a node *i* belongs to the set of upper nodes if its depth is lesser or equals to half of the network’s depth and belongs the set of lower nodes otherwise.

Right after the completion of the deployment phase, the hand shaking process begins among nodes. Nodes broadcast a control packet in their surroundings that includes its ID, residual energy and depth information. Surrounding in-range nodes of the source node receive the packet and check for eligible neighborhood criterion which is as follows:

**Rule** **2:** 
(10) $$j\;\epsilon \left\{\begin{array}{c} \hat{N_{i}}\;if\;i\;\epsilon \;N_{U}\;and\;d\left(i\right)\leq d\left(j\right)\;or\;if\;i\;\epsilon \;N_{L}\;and\;d\left(i\right)>d\left(j\right) \end{array}\right.$$

This rule states that *j* belongs to the neighboring list of *i* if the following conditions are fulfilled. Stated as:

- *i* belongs to the upper node’s list and depth of *i* is less than the depth of *j*.
- *i* belongs to the lower node’s list and depth of *i* is higher than the depth of *j*.

**Figure 4:**
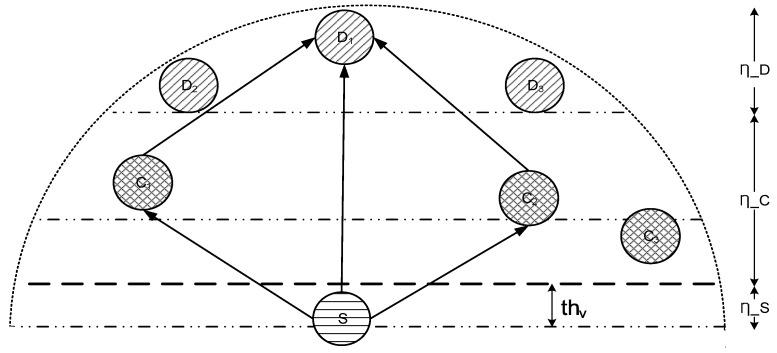
Slicing of transmission range of source node.

For convenient data transmission process and energy conservation, transmission range of the source node is divided into three slices such that the first slice contains source node itself and possible cooperating nodes $\eta_{c}$ , the second slice also contains possible cooperating nodes $\eta_{c}$ (see Figure 4 whereas, the third slice contains $\eta_{d}$ only.

It is stated that neighboring node of a source node consists of possible cooperating and destination nodes $\hat{N_{i}}=\eta_{c}+\eta_{d}$ . This rule qualifies source node to transmit data such that only eligible cooperating nodes receive and compute that packet whereas eligible destination nodes discard it immediately and thus conserves energy. In the reference of *i* as a source node now consider a node *j* that may belong to $\eta_{c}$ or $\eta_{d}$ depending upon the conditions written as follows:

(11) $$j\;\epsilon \left\{\begin{array}{c} \eta_{c}\;if\;d_{th}^{\alpha }<\left|d_{j}-d_{i}\right|<d_{th}^{\beta }\;\forall \;\;i,j\;\epsilon \;N\\ \eta_{d}\;if\;\left|d_{j}-d_{i}\right|>d_{th}^{\beta }\;\forall \;\;i,j\;\epsilon \;N \end{array}\right.$$

where $d_{th}^{\alpha }$ and $d_{th}^{\beta }$ (positive real numbers) are two thresholds that change according to current node density. Regarding nodes’ density two possibilities arise here;

- Node density is greater than the marginal value,
- Nodes density drops to the marginal value.

Whereas, marginal value is equivalent to half of the total number of deployed nodes.

Moreover, as stated earlier, the threshold values are dynamic in nature therefore; initially the difference between the two thresholds is kept low so that lesser nodes fulfills the eligible neighborhood criterion. This causes energy conservation as lesser nodes receive the information being sent by the source node. However, with the passage of time nodes deplete their energies and at that instant keeping lower difference among thresholds would not be a good approach. This is due to the fact that the source node is unable to find sufficient neighbors around and remains unattended. This situation could lead to a degraded reliability factor due to fewer or non availability of cooperating nodes. Hence, as a solution of sparsity we keep on increasing the threshold difference. Therefore, our proposed model is adaptive in this aspect. Mathematically,

(12) $$\left\{\begin{array}{c} d_{th}^{\alpha }-d_{th}^{\beta }>\;\delta \;\;\;\;if\;N^{\prime }\;\leq \;1/3\;\left(N\right)\\ \Omega \;<\;d_{th}^{\alpha }-d_{th}^{\beta }<\;\delta \;if\;N^{\prime }\;>\;1/3\;\left(N\right) \end{array}\right.$$

where, *ω* and *δ* are real numbers.

For our proposed model, another important feature is the GWs involvement. GWs are nodes with relatively higher initial energy levels as compare to MNs. This is because of the reason that in our technique, each node transmits its own data packet as well as the data packets being generated by the predecessor nodes. Since data forwarding burden is highest on the chain heads which causes their rapid early energy depletion and leads to shorter network lifetime and stability period. Moreover, AUV also assists the process while moving on its predefined trajectory and collecting and forwarding data from GWs towards sink. AUV ensures data communication at minimized distance leading to network lifetime prolongation and it takes stops at specific positions known as sojourn locations for specific time called sojourn time.

## 7. Relays Selection

Relays selection is another basic concern of our proposed protocol since proper relays selection immensely contributes in network’s enhanced performance. Multiple protocols have been suggested up-till now that incorporates relay selection method to achieve specified parameters considering different factors e.g., number of alive neighbors, node’s energy, node’s depth and others. However, as our main objective is the network’s energy conservation and data reliability, therefore, considering these parameters our protocol selects relays by keeping in view the channel characteristics and depth information of nodes. Furthermore, in order to ensure successful packet acceptance at the destination, all nodes are enabled to demand for two retransmissions of a single received packet. This is done via transmitting NACK1 and NACK2 in consecutive phases.

A new set $B_{c}$ is generated by the following steps:

- Sender nodes broadcast the packet in their respective transmission ranges.
- Nodes residing in the list of possible cooperation set takes part in cooperation process one by one.
- If BER exceeds the threshold value , receiver sends NACK message towards the sender node as well as the source node.
- Source node updates the history of wireless link quality based on the number of retransmission requests.
- We assume a slow and flat Rayleigh faded environment.
-
$B_{c}$ is updated occasionally depending upon the weight updates carried out for that specific path.
- In this way multiple cooperating nodes are available to cooperate with the sender node.

**Figure 5:**
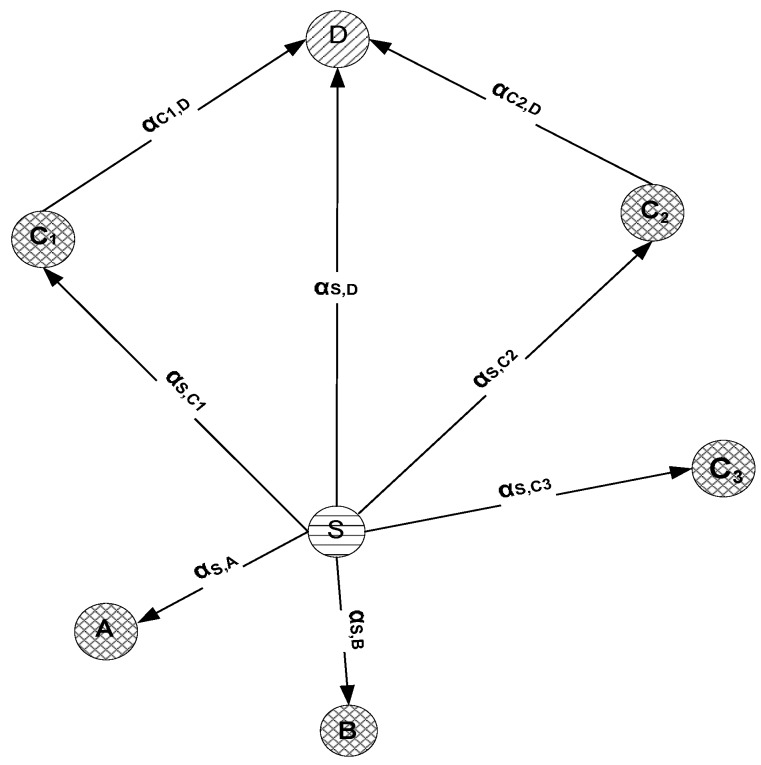
Cooperative diversity system.

In our suggested technique, relay communication takes place in two stages as depicted in Figure 5. In the first stage, the source node broadcasts whereas cooperative and destination nodes receive that packet. Upon reception, the cooperative node uses AF to relay the received data. In comparison to DF, AF requires reduced E2E delay as the relay node operates time-slot by time-slot [43]. Moreover, AF demands lesser computational powers because of absence of decoding and quantizing at the relay node. In secondary stage, this processed data is re-forwarded towards the destination. Upon reception, the destination node applies the Maximal Ratio Combining technique (MRC) over the entire copies of received packets. MRC is adopted because it is the optimum combiner for independent AWGN channels. MRC functions by adding the signals such that different proportionality constraints are employed for each channel.

Suppose $\eta_{c}$ consists of two nodes ( $C_{1}$ and $C_{2}$ ), *x* is the original message signal being transmitted and AF is used as a cooperative relay model, then:

(13) $$S_{S,D}\left(t\right)=\alpha_{S,D}\;x\left(t\right)+n_{S,D}\left(t\right)$$

(14) $$S_{S,C_{i}}\left(t\right)=\alpha_{S,C_{i}}\;x\left(t\right)+n_{S,C_{i}}\left(t\right),\;C_{i}\;\epsilon \;\eta_{C}$$

(15) $$S_{C_{i},D}\left(t\right)=\alpha_{C_{i},D}\;x\left(t\right)+n_{C_{i},D}\left(t\right),\;C_{i}\;\epsilon \;\eta_{C}$$

Considering the above equations, $S_{S,D}\left(t\right)$ be the representation of a signal being sent from *S* and received at *D*. Whereas, $S_{S,C_{i}}$ and $S_{C_{i},D}$ be the signals sent from *S* towards $C_{i}$ and from $C_{i}$ towards *D* respectively. Moreover, $\alpha_{S,D}$ and $n_{S,D}$ be the channel gain and channel noises existing on the link in between *S* and *D* whereas $\alpha_{S,C_{i}}$ and $n_{S,C_{i}}$ be the channel gain and channel noises in between *S* and $C_{i}$ respectively. Similarly, $\alpha_{C_{i},D}$ and $n_{C_{i},D}$ be the channel gain and channel noises existing at the $C_{i}$ and *D* link.

In order to mitigate the impact of fading over the received signal, receive diversity is employed in our protocol. Thus, the probability that all the copies of that signal get faded is minimized. Initially, nodes broadcast control packets and maintain the neighboring list. Soon after, the destination and cooperative nodes are finalized (Algorithm 1).
**Algorithm 1 : RE-AEDG**1:**procedure:**
*NODE-INITIALIZATION*2:*MinorAxis* ← *b*3:MajorAxis ← 2b4:*CentralPoints* ← 
$x_{0}$
, 
$y_{0}$
5:*Energy(MemberNodes)* ← *μJ*6:**return** TRUE7:**end procedure**8:**procedure:** GATEWAY-NODE-SELECTION9:*GWsNodeliesInTheRangeOfSinkNode*10:**if**
*Node.GWs* = *TRUE*11:*E(GWsNode)* ← *E(MemberNodes)* + *ζ*12:**return** TRUE13:**end procedure**14:**procedure:** NEIGHBOR-FINDING-MODULE15:**if**
*d(i)* > 
$\frac{1}{2}\left(LengthOfNetwork\right)$

*and d(i)* > *d(j)*16:*ϕ* ← 
d(i)−d(j)
17:**elseif**
*d(i)* < 
$\frac{1}{2}\left(LengthOfNetwork\right)$

*and d(j)* > *d(i)*18:*ϕ* ← 
d(j)−d(i)
19:**end if**20:**if**
*ϕ* ≤ *TxRange*21:*NeighborFound* = *TRUE*22:**return** TRUE23:**end procedure**24:**procedure:** NEXT-HOP-FINALIZATION25:*ϑ* = *and (Neighbor, GWsNode)*26:**if**
*SumOf(ϑ)* ≥ *1*27:*NextHop* ← *MinimumOfDownNeighbors(ϑ)*28:**elseif**
*SumOf(ϑ)* ≤ *1*29:*NextHop* ← *MinimumDepthNeighbor*30:**elseif**
*ϑ* = = *FALSE and NodeIsGWsNode*31:*NextHop* ← *Sink*32:**end if**33:**return** TRUE34:**end procedure**35:**procedure:** BIT-ERROR-CHECK36:**if**
*BEROfPacket* ≤ *ThreshholdBER*37:*PacketAccepted*38:**elseif**
*BEROfPacket* ≥ *ThreshholdBER*39:*PacketDiscarded*40:**return** TRUE41:**end procedure**42:**procedure:** PACKET-RESEND-REQUEST43:**if**
*BEROfPacket* ≥ *ThreshholdBER*44:**if**
*PacketResendRequest* == *0 or 1*45:*SendPacketResendRequest*46:*PacketResendRequest* = *PacketResendRequest* + *1*47:**end if**48:**end if**49:**end procedure**


The source nodes broadcast sensed data within their transmission ranges. The receiver node checks the BER of the packet; if it exceeds the threshold BER (0.5 out of 1) then it drops the packet immediately and asks the sender node for re-transmission. If it is not the case then the cooperative nodes apply the AF mechanism and re-broadcast the packet. As soon as the destination nodes receive the required copies of packet, these apply the MRC technique. After the required processing, the destination node re forwards the packet. This process continues till data reaches the AUV.

## 8. Sink Trajectory

Successive retransmissions in cooperative routing lead to excessive energy consumption of the network nodes, which is unfavorable due to limited battery capacity of nodes. In order to mitigate this issue, we use mobile sink which moves on an elliptical trajectory [44]. AUV cooperates with MNs and GWs such that it goes to the near locality of GWs, receives the data packets and conveys them towards the surface sink. In this way, the overall communication distance is minimized. We consider an elliptically trajectory with horizontal major axis, such that;

(16) $$\frac{{\left(x-x_{o}\right)}^{2}}{M_{AUV}^{2}}\;+\;\frac{{\left(y-y_{o}\right)}^{2}}{m_{AUV}^{2}}\;=\;1$$

and;

(17) $$M_{AUV}\;=\;2\;\times \;m_{AUV}$$

**Figure 6:**
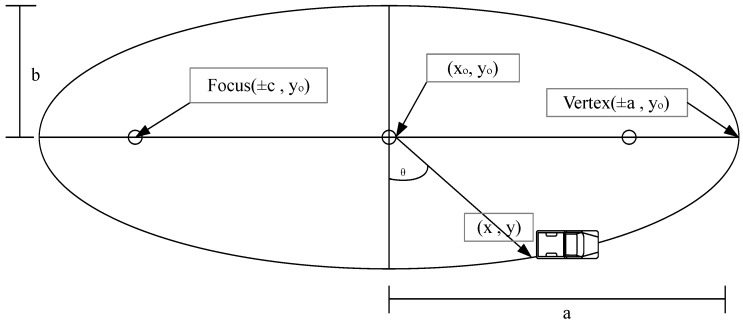
Sink mobility pattern.

Foci points for non centered ellipse are defined at $\left(\pm c,y_{o}\right)$ and $\left(\pm c,y_{o}\right)$ and vertices of ellipse are at $\left(\pm a,y_{o}\right)$ and $\left(\pm a,y_{o}\right)$ whereas, $c\;=\sqrt{M_{AUV}^{2}\;-\;m_{AUV}^{2}}$ and *x* and *y* show the running time coordinates of AUV and they are scaled using the following equations:

(18a)x=xo+MAUVCos(θ)(18b)y=yo+mAUVSin(θ)

where *θ* is the angle that AUV makes with the center of ellipse as shown in Figure 6.

## 9. RE-AEDG: Optimization Models

In this section, we discuss an energy consumption model and a delay optimization model to overcome the limitations of cooperative communication technique and mobility assisted routing.

### 9.1. Delay Minimization Model

Our proposed RE-AEDG implements cooperative communication to improve reliability while paying the cost of energy consumption and data latency. After energy consumption minimization model in the previous sub section, we present delay minimization model in this portion of paper.

(19) $$\text{Min}\sum_{i=1}^{n}Dy_{i}\;\forall \;i\epsilon N$$

Subject to:

(19a)dth≤dihi≤TxR∀iϵ(MNs||GWs)(19b)0≤hi≤hmax∀iϵMNs(19c)dihi≥diηi(19d)tmin≤St≤tmax(19e)0≤ρhi≤ρth∀iϵMNs(19f)0≤Hi≤Hmax∀iϵ(MNs||GWs)whereasH=1Re×dihi(19g)Pi(t)≤Ccap∀iϵ(MNs||GW)(19h)MAUV≥13Wnet

Our objective in Equation (19) is to minimize the time delay created in data transmission process. Equation (19a) defines that depth difference between *i* and its next hop $h_{i}$ must remains in between depth threshold and predefined transmission range. Whereas depth threshold is kept to a value, such that sender node may not be able to define its priority node at an extremely near vicinity. Applicability of this law causes larger depth difference between transmitter and receiver which in turn results in greater energy consumption and lowered delay. This can also be written as *D* ∝ *No.o f hops*. Equation (19b) indicates that the total no. of hops a packet requires to reach destination must not exceed than the maximum allowable no. of hops. Implementation of this clause prevents extra per packet network energy consumption. Equation (19c) depicts that depth difference in between *i* and $h_{i}$ must be maximum out of Eligible Neighbors set. This rule in incorporated because transmission at lower distance node increases packet transmission reception rate. Equation (19d) states that sojourn stop time for an AUV must be an optimum duration. Failing to implement this constraint causes GWs to hold packets for larger time which causes extensive delays. Equation (19e) shows that if a node has lesser number of predecessor nodes then queuing delay for packet sufficiently reduces which in turn leads to lowered processing delays. For the purpose of delay optimization Equation (19f) suggests that holding time of a packet must be an optimum value. Moreover, it also states that nodes with higher residual energy have lower holding time. Therefore, lowered energy nodes also prevents them from data transmission by holding this specified clause. Equation (19g) indicates that packets being generated by *i* at time *t* must not exceeds the channel capacity. This clause mitigates the channel congestion possibilities and lowers overall packets drop rate. Implication of this law lowers data re-transmissions probability. Equation (19h) defines that major axis of sink trajectory must cover at least one third portion of the network width, this causes reduced transmission distance between source and destination nodes.

### 9.2. Energy Consumption Minimization Model

In order to improve PAR at the destination, our proposed model incorporates cooperative communication technique, however; at the cost of excessive energy consumption. Thus, our objective to minimize the energy consumption cost is formulated as follows:

(20) $$\text{Min}\sum_{i=1}^{n}E_{i}\;\forall \;\;i\epsilon N$$

Subject to:

(20a)0≤ρhi≤ρth(20b)hi=zifdi,z≤TxR∀iϵMNs&zϵGWsjifdi,z≥TxR∀i,jϵMNs&zϵGWs(20c)Rehi≥ReNi^∣hiϵwidehatNi∀iϵMNs(20d)Pemin≤Pe≤Pemax(20e)hz=AUV∀zϵGWs

Our objective in Equation (20) is to minimize the overall network energy consumption. Equation (20a) indicates that the number of predecessor nodes of the selected hop of *i* should be less than or equal to the predefined threshold predecessors. Therefore, it causes equal data flow burden among nodes. Violation of this rule can cause an extensive data flow at a node and may result in enhanced energy depletion and a shorter network stability period. Equation (20b) illustrates that if *z* is a GW and it lies inside transmission range of a sender node *i* then *z* would be a preferred node. Hence, number of hops a packet takes to reach to the destination are lowered resulting is lesser energy consumption is single packet transmission process. Equation (20c) provides the the balanced energy consumption among nodes. It depicts that next hop of *i* should have maximum residual energy out of the Eligible Neighborhood set of a node. Equation (20d) defines that per packet energy consumption $P_{e}$ should be an optimum value, provided the upper and lower bounds $P_{e}^{min}$ and $P_{e}^{max}$ of per packet energies. This energy bound limits transmission distance among communicating nodes. Equation (20e) states that for *z* being a GW the next hop is always an AUV and no inter-hop communication exists between the two GWs. Failing to implement this clause can enhance the number of retransmissions of data packets and as a result an increased energy consumption.

## 10. Applications of Proposed Methods

The proposed model can be used for most of the applications that can tolerate additional delays however, requires lesser average energy consumptions and desires the network to remain operational for longer duration of time e.g., Oceanography, seismic predictions, pollution detection, oil/gas field monitoring and others. Moreover, implementation of our proposed architecture is also feasible for data sensitive applications such as tactical surveillance and navigation, etc.

Along with all the above mentioned applications our proposed energy consumption optimized version is also cost effective because it performs long term aquatic monitoring and requires lesser replacement and recharging of batteries. Besides that delay optimized method has better applications where data provision at central unit is desired in lesser time while insuring information reliability such as earthquake sensing, tsunami detections, military applications and others.

## 11. Simulation Results and Discussions

In this section, we assess the performance efficiency of RE-AEDG in comparison of other two protocols; U-LVRP and AEDG. LVRP is a protocol designed for terrestrial WSNs. It is chosen because its layering layout resembles our schematic in many aspects. We have simulated LVRP in underwater environment (U-LVRP). In the simulations we considered a 500 m × 500 m network field. The other selected simulation parameters are depicted in Table 3.

We chose the following performance matrices.

- **Network lifetime**: Time in which network is considered to be operational. i.e., from network configuration till the death of last node. It is calculated in seconds.
- **Stability period**: Time (in second) from network establishment till the death of last node.
- **PAR**: It is the ratio of total number of packets reached at the sink to the number of packets being sent by sink in specified interval.
- **Throughput rate**: Throughput is defined as the number of packets successfully reached the water surface sink. It is measured in packets per second.
- **E2E delay**: It is the time taken for a packet to be transmitted across a network from source to destination.

### 11.1. Results and Discussions

The discussions are divided along two sub sections where the originally designed protocols are examined along with their delay and energy optimized versions.

### 11.2. Non Optimized and Delay Optimized Protocols

This portion evaluates the performance of non-optimized in contrast to delay optimized protocols. Plots and their discussions are as follows.

#### 11.2.1. Throughput and PAR

**Figure 7:**
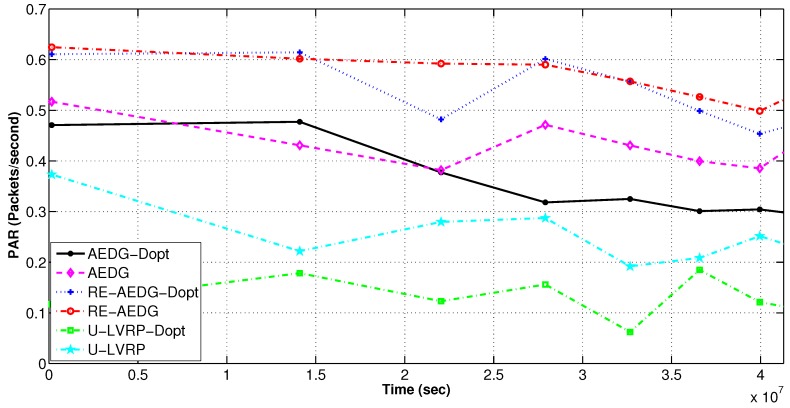
PAR in delay optimized and non optimized protocols.

**Figure 8:**
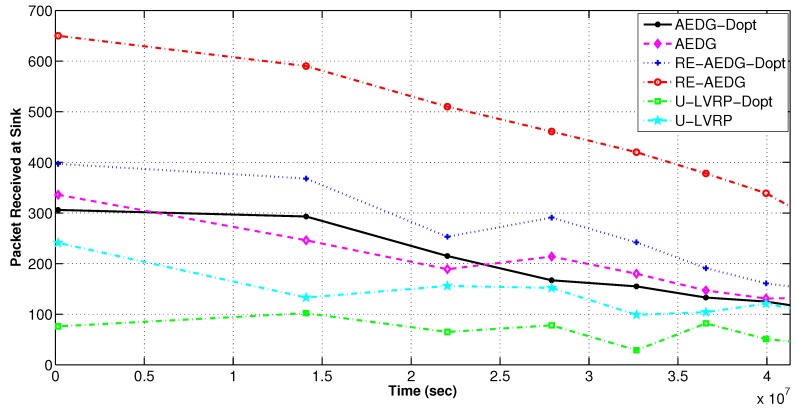
Packets received at sink in delay optimized and non optimized protocols.

RE-AEDG has maximum throughput both in optimized and non optimized versions. In RE-AEDG, presence of GWs at both sides of AUV and sink trajectories highly influence the number of packets that successfully reach sink in a given time interval. In RE-AEDG, AUV comes in the near vicinity of the nodes causing the GWs to be selected nearer the MNs. As a result, the number of hops and the required re-transmissions decreases significantly. In addition to that shorter communication routes also lower the probability of data collision and causes the loss of sensitive information; as shown in Figure 7 and Figure 8.

In the figures, AEDG shows an intermediate state PAR due to the fact that it does not involve cooperative routing mechanism in its transmission process. Hence, it has lowered number of packets received at sink and PAR as compare to RE-AEDG. Whereas; for U-LVRP random movement of AUVs demand the nodes to dynamically define their communication paths. Therefore, a relatively higher packet drop rate is observed for this scheme as compare to the other protocols.

Furthermore, the implication of $d^{th}$ is also visible in the optimized version of the schemes in terms of their lowered PAR and the number of packets being received at sink. $d^{th}$ reduces the size of eligible neighbourhood set for sender nodes and sometime causes them to stand alone in network specifically when the network becomes sparse.

#### 11.2.2. E2E Delay

**Figure 9:**
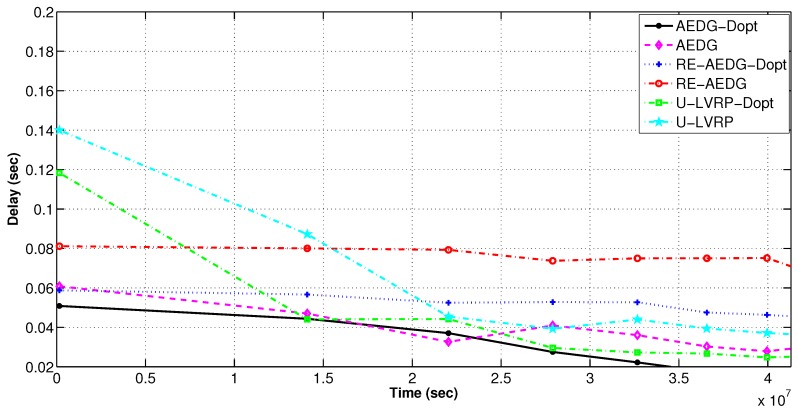
Message latency in delay optimized and non optimized protocols.

From Figure 9, it is clear that the delay optimized versions of the protocols show lower delay than their respective non-optimized versions. In both versions of AEDG and RE-AEDG, the GWs are the major cause of delay. RE-AEDG-Dopt shows the highest computational and holding delays as the nodes in RE-AEDG carry out the cooperation.

Whereas, U-LVRP’s random AUV motion causes an increased overhead on the nodes to dynamically select their routing tracks. At every sojourn stop, anchor node reselection also results in higher computational delays. Thus these are the major delay creation factors for both versions of U-LVRP. The steep behavior for both curves of U-LVRP is because they initially lead to rapid network sparseness. Sparseness influences delays because nodes need to compromise their priority for neighbor node selection. However, with the passage of time nodes in U-LVRP start energy conservation causing rapid reduction in delays as compared to the other two protocols.

#### 11.2.3. Death Rate of Nodes and Rate of Energy Utilization

**Figure 10:**
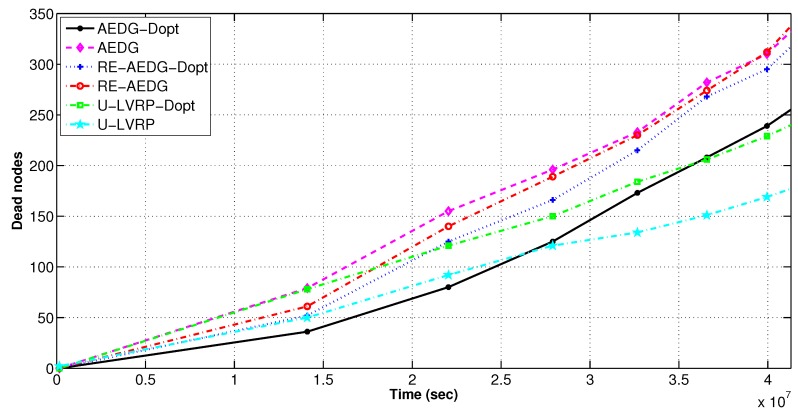
Death rate of nodes in delay optimized and non optimized protocols.

In Figure 10, AEDG shows rapid sparseness due to absence of optimized AUV trajectory. Moreover, for AEDG no restriction is implemented on the number of predecessors that can be attached to a node. Therefore, over burdened nodes discharge their batteries earlier causing predecessors to search for some other non feasible cooperating node. This causes nodes’ energy depletion at an earlier stage.

**Figure 11:**
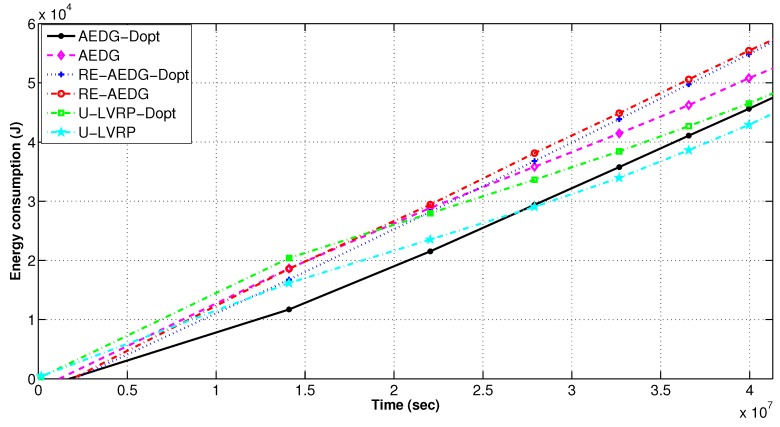
Energy consumption in delay optimized and non optimized protocols.

Figure 11 shows that despite cooperative routing in RE-AEDG, AEDG initially shows comparatively equivalent energy dissipation rate, however, afterwards it depicts comparatively reduced network energy consumption. This happens because of the AUV trail in RE-AEDG: it employs an extended AUV trail so that it may select the GWs from both sides of its trajectory covering more number of MNs and supporting them in data gathering and communication phase. Later on the reduced energy consumption of AEDG is because of the fact that with the passage of time its packet drop rate increases (Figure 8) causing lesser packet forwarding burden on the intermediated nodes and consequently lesser network energy consumption. AEDG also remains with lower alive number of nodes causing per instance reduced energy consumption.

AEDG-Dopt shows the minimum energy consumption because of optimized version of sink trajectory and predecessors attached to a specific node. On the other hand, U-LVRP consumes relatively higher energy. In-spite of random motion of sink U-LVRP-Dopt still shows reduced energy consumption. Delay optimization also leads to lower energy consumption in AEDG and RE-AEDG because the number of predecessors attached to a node (Equation (19e) and Equation (19b)) that restricts the maximum number of hops a data packet travels form source to destination. Figure 10 also strengthens our claim by depicting the relative number of nodes died among different protocols per interval of time.

### 11.3. Non Optimized and Energy Optimized Protocols

This portion analyzes the energy optimized and non optimized versions of the selected protocols. Plots and their discussion are presented as follows.

#### 11.3.1. Number of Alive Nodes and Network Energy Consumption

**Figure 12:**
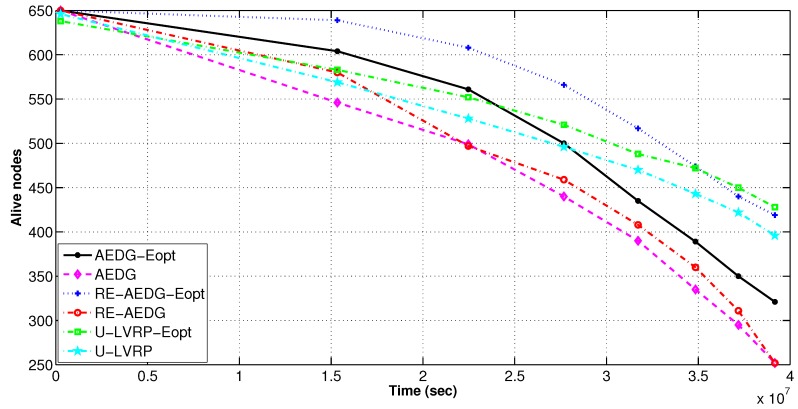
Number of alive nodes per interval in comparison to energy optimized protocols.

**Figure 13:**
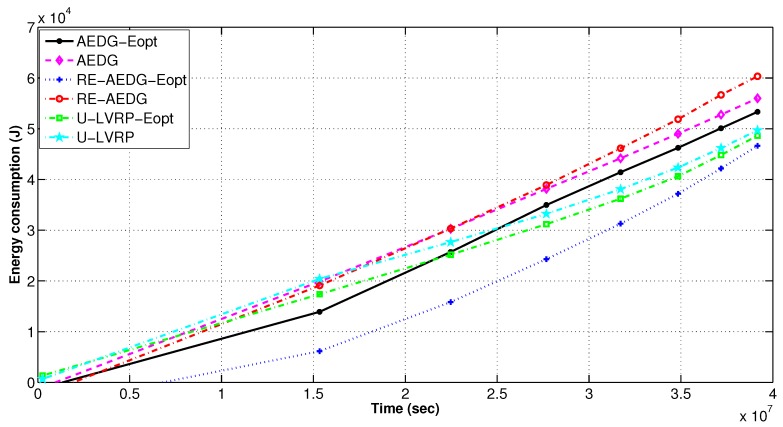
Energy consumption in energy optimized and non optimized protocols.

In RE-AEDG, the defined sink trajectories and higher number of GWs lead to prolonged network lifetime. Initially both versions of U-LVRP protocol show less difference in energy consumption but with the passage of time the difference gets higher. This is due to the fact that initially nodes find their neighbors in their vicinity. However, with the passage of time the number of nodes in the network decreases. Ultimately, increased transmission distance between the neighboring nodes results on higher energy consumption of the sender node. On the other hand, for optimum RE-AEDG and AEDG, nodes create links by updating the number of predecessors already attached with the neighbor under consideration. This check reduces the packet reforwarding burden on the nodes. Eventually, the energy consumption is balanced in the network. Avoidance of intercommunication between the GWs leads to enhanced stability period as shown in Figure 12 and Figure 13. In RE-AEDG-Eopt, the lowest energy consumption depicts that the cost being payed for achieving higher throughput rate could be minimized through controlled optimization process.

#### 11.3.2. Packets Loss and PAR

**Figure 14:**
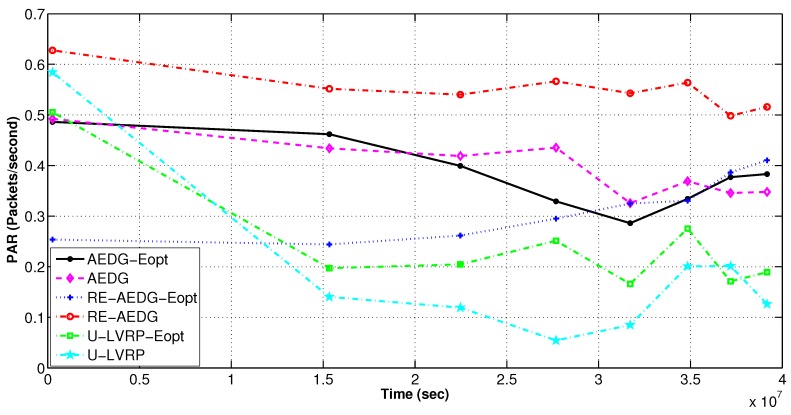
PAR of networks in energy optimized and non optimized protocols.

**Figure 15:**
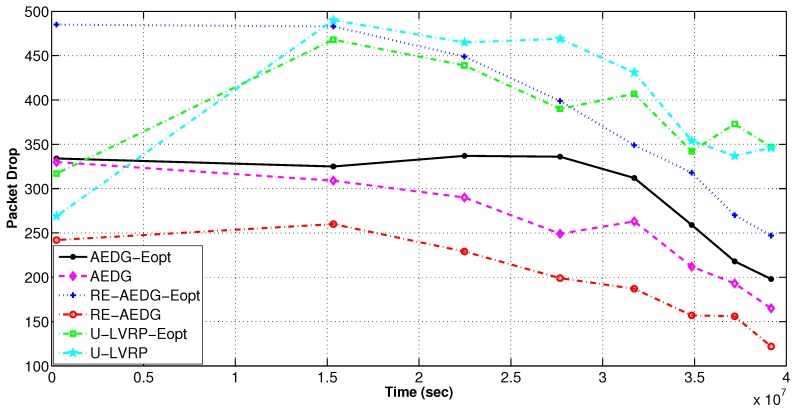
Packets drop rate in energy optimized and non optimized protocols.

To minimize energy consumption we employ a constraint Equation (20a) that limits the predecessors attached to every node. However, there exists a tradeoff between energy utilization and packet drop rate. It is to note that, although energy remains conserved in optimized protocols but at the cost of high packet drop rate due to inability of sender node to find the next hop node. RE-AEDG shows the minimum packet drop rate because AUV itself comes in the vicinity of nodes and shortens their communication path. Eventually, lowered packet collision rate and improved reception of data at destination. Additionally; more the number of packets being generated more is the possibility of successful packet reception at sink. Since the packet drop rate is measured only for alive nodes, therefore; the down fall of the packet drop curves in Figure 14 and Figure 15 shows the rate at which the network becomes sparse.

#### 11.3.3. E2E Delay

**Figure 16:**
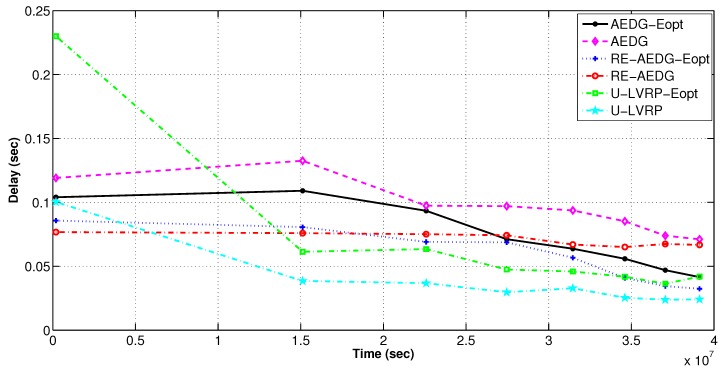
E2E delay in energy optimized and non optimized protocols.

In the Figure 16 it is visibly shown that both versions of AEDG have induced higher E2E delay as compared to RE-AEDG. This is due to the fact that data reaching at their respective gatherer stays there for the time until AUV approaches to them. Moreover for AEDG, AUV track is smaller as compare to RE-AEDG so; the intercommunication distance between the source and destination node (AUV) is enlarged. Whereas for RE-AEDG, threshold based neighbors assignment to a node leads to comparatively reduced queuing delay. This is because it specifies the number of predecessor nodes that could be attached to another node. Thus, the E2E delay of RE-AEDG is relatively lesser than both versions of AEDG. However, despite of the random movement of AUVs in U-LVRP the presence of two of mobile sinks normalizes the additional message latencies created by dynamic selection of communication routes between nodes and AUVs.

## 12. Performance Tradeoffs

AEDG provides a good throughput rate due to the involvement of GWs and AUV. AUV itself visits the GWs and gathers data from them, thereby, increasing the throughput. This throughput improvement in AEDG is achieved at the cost of higher energy consumption and greater message latency. Both are caused by predefined but non optimized trail followed by AUV in the protocol. On the other hand E2E delay for AEDG is minimized by applying a set of constraints but for AEDG-Dopt the considered network shows degraded PAR. Implication of $d^{th}$ is visible here. It reduces the number of hops that a packet takes while reaching to sink but it also reduces the eligible neighbors for the sender node. Similarly AEDG-Eopt performs better in terms of energy conservation at the cost of low PAR. For energy optimization Equation (20a) is applied which limits the number of predecessors attached with a node. This saves computational powers at receivers but highly effects the PAR.

RE-AEDG shows better throughput but depicts higher delays in data packet arrival at the sink. One of the two reasons is the involvement of cooperative communication mechanism. Whereas, the other is that the GWs hold data for more amount of time causing the extended E2E delays. To suppress the above mentioned deficiency, we optimized the RE-AEDG scheme for minimized delay but it pays the cost of lowered packet delivery at the sink. In delay optimization, $d^{th}$ reduces the network performance in terms of data throughput. Similarly, RE-AEDG-Eopt conservers the overall network energy but enhances the packet drop rate. This is due to the restricted number of hops a data packet could avail on its one way path.

Two AUVs that aid MNs and GWs in data transmission phase causes them to remain alive for a longer duration. However, in spite of this stated argument the ULVRP’s performance is still the worst in terms of throughput achievement. This is because the AUVs that take random sojourn stops, lead towards an interference in the voronoi scope of each other. Their random movement causes repeated selection and reselection of data path for nodes hence, reduced data arrival rate at sink. ULVRP-Dopt achieves advancement in delay at the cost of increased energy consumption because $d^{th}$ assigned to nodes causes the hops at farther distance.

RE-AEDG has higher PAR due to incorporation of cooperative communication. RE-AEDG-Eopt has lesser E2E delay because queues for every node are limited in this case. The AEDG has better throughput at the cost of delay. A comprehensive over view of performance tradeoffs is shown in Table 4.

## 13. Conclusions

In this paper, we have presented the RE-AEDG protocol for UWSNs. The RE-AEDG employs the cooperative routing scheme to improve reliability of data. However, the introduced cooperative routing leads to high energy consumption of nodes. In order to solve this problem, mobile sink is used for data gathering. Also, to further reduce the cost being paid, two optimized versions of RE-AEDG are presented catering the issues of high energy consumption and high E2E delay. Simulation results demonstrate that RE-AEDG has improved data reliability as compared to AEDG and U-LVRP. In the terms of energy consumption and message latency, optimized versions of RE-AEDG perform better than the selected existing techniques.

RE-AEDG induces additional message latencies, comparative to the counterpart schemes. Since relay selection highly affects the network performance, therefore, a more comprehensive relay selection model to overcome this limitation is under consideration. Moreover, the sink mobility and sojourn time for AUV can be optimized further to reduce the E2E delay.

## Figures and Tables

**Table 1:** Comparison of the state-of-the-art work.

Technique/Ref	Objective(s)	Achievement(s)	Deficiency(ies)	Field/Architecture
C- ARQ [6]	i- Provides alternative routing paths ii- Retransmission of erroneous data	i- Exploits broadcast nature of transmissions for throughput efficiency	i- Higher energy consumption ii- High E2E delay iii- Lower packets acceptance rate iv- Network partition problem	i- 2D-UWSN ii- Two terminal cooperative relay model
SRC DNC [7]	i- Best relay node selection ii- Data authenticity iii- Minimized retransmissions	i- Enhanced data integrity ii- Improved packet reception rate	i- In-efficient energy usage ii- High E2E delays iii- High protocol overhead	i- 2D-UWSN ii- Random deployment of nodes
COBRA [8]	i- Best relay selection ii- Overcome frequency selective fading iii- Minimized OPT time	i- Throughput improvement ii- Reduced delay if relay is selected for minimized OPT time iii- Minimized packets collision	i- High E2E delay, if packet size is small ii- Increased energy consumption iii- Network partitioning	i- 2D-UWSN ii- Random deployment
[9]	i- Relay selection based on propagation delay and SNR ii- Data authenticity	i- Improved delay induction ii- Improved bit error rate	i- Reduced stability period ii- Reduced network lifetime iii- Efficient energy utilization	i- 2D-UWSN ii- Random deployment of nodes
DEADS [10]	i- Data reliability ii- Throughput iii- Energy efficiency iv- Adaptive routing	i- PAR ii- Reduced packet drop rate iii- Reduction in energy consumption iv- Optimized *d_th_*	i- High E2E delays ii- Control and processing overhead iii- Stability period compromized	i- 3D-UWSN ii- Randomly deployed iii- Two mobile sinks
AEDG [37]	i- Reduced as well as balanced energy consumption ii- Efficient data gathering	i- Improved stability period ii- Prolonged network lifetime iii- Comparatively reduced packet drop rate	i- No significant raise in data authenticity ii- Message latency iii- Control packet overhead	i- 2D-UWSN ii- Heterogeneous randomly deployed nodes iii- One AUV
LVRP [36]	i- Efficient distributed data routing ii- Reduced routing update frequency iii- Energy conservation	i- Alleviated energy hole problem ii- Improved packet delivery ratio iii- Less forwarding overhead	i- Path selection overhead due to random movement of AUV ii- AUVs leave field un-attended and penetrate in each another voronoi scope iii- Long communication paths	i- 2D-UWSN ii- Uniform random circular deployment of nodes iii- Two to six number of AUVs

**Table 2:** Attributes of types of deployed nodes.

Attributes	MNs	GWs	AUV
Modem	Acoustics	Acoustics	Acoustics, Radio
Power	Less	High	Not a constraint
Quantity	Larger	Smaller	One
Interacts with	MNs, GWs	MNs, GWs, AUV	GWs, Sink
Info. sensing	Yes	Yes	No
Device mobility	No	No	Yes

**Table 3:** Simulation specifications for RE-AEDG.

Parameter	Values
Network field	500 m^2^
Nodes deployed	650
Number of GWs	100
Number of MNs	550
Per node initial energy of members	100 J
Per node initial energy of GWs	115 J
Transmission range	70 m
Packet size	50 bytes
Number of AUVs for U-LVRP	2
Number of AUVs for AEDG and RE-AEDG	1

**Table 4:** Performance tradeoffs.

Protocol	Enhancements Achieved	Figure	Price Paid	Figure
AEDG	Throughput	8	Energy consumption	11
AEDG	Throughput	14	E2E delay	16
AEDG-Dopt	E2E delay	9	PAR	7
AEDG-Dopt	Energy consumption	10	PAR	7
AEDG-Eopt	Energy utilization	13	PAR	14
AEDG-Eopt	E2E delay	16	PAR	14
RE-AEDG	Throughput	8	E2E delay	9
RE-AEDG	PAR	7	E2E delay	9
RE-AEDG-Dopt	E2E delay	9	Throughput	8
RE-AEDG-Eopt	Number of alive nodes	12	Packet drop rate	15
ULVRP	Energy consumption	11	PAR	7
ULVRP-Dopt	E2E delay	9	Energy consumption	11
ULVRP-Eopt	Energy consumption	13	Packet drop rate	14

## Abbreviations

WSNs	Wireless sensor networks
UWSNs	Underwater wireless sensor networks
AUV	Autonomous underwater vehicle
C-ARQ	Cooperative automatic repeat request routing protocol
SRC	Selective relay cooperation
DNC	Dynamic node cooperation based protocols
COBRA	Cooperative best relay assessment
EASR	EASR
(*ACH*)^2^	Adaptive clustering habit
CoUWSN	Cooperative Energy-Efficient Protocol for Underwater WSNs
BTM	Balance Transmission Mechanism
SEECH	Scalable Energy Efficient Clustering Hierarchy
EDIT	Energy Delay Index for Trade-off
LAFR	Link-state based Adaptive Feedback Routing for UASNs
IAMCTD	Improved Adaptive Mobility of Courier Nodes in Threshold-Optimized DBR
AURP	An AUV-Aided Underwater Routing Protocol
SPARCO	Stochastic Performance Analysis with Reliability and Cooperation for UWSNs
LESCA	Location-Energy Spectral Cluster Algorithm
WDFAD-DBR	Weighting depth and forwarding area division DBR
BLM-MCT	Boundary detection for large-scale coverage holes in WSN based on minimum critical threshold constraint
GAECH	Genetic Algorithm Based Energy Efficient Clustering Hierarchy in WSNs
GPNC	Geographic and partial network coding based routing protocol for UWSNs
DEADS	Depth and energy aware dominating set based algorithm for cooperative routing along with sink mobility
AEDG	An efficient data-gathering routing protocol
LVRP	Layered voronoi scoping-based routing protocol
RE-AEDG	Reliable AUV-aided Efficient Data Gathering Routing Protocol for UWSNs
Dopt	Delay optimized
Eopt	Energy optimized
AF	Amplify and forward
DF	Decode and forward
MRC	Maximal ratio combining technique
ERC	Equal ratio combining technique
GWs	Gateways
MNs	Member nodes
NACK	Negative acknowledgement
Dim	Dimensional
BER	Bit error rate
PAR	Packets acceptance ratio
SNR	Signal to noise ratio
E2E	End to end
*I_tran_*	Acoustic intensity at transmitter
*I_receiver_*	Acoustic intensity at receiver
*R*	Distance between transmitter and receiver (m)
*a_u_*	Absorption coefficient
*f*	Frequency (KHz)
*k*	Spreading factor
*N_s_*	Shipping noise
*N_w_*	Wind noise
*N_t_h*	Thermal noise
*N_t_*	Turbulence noise
*S*	Source node
*C*	Cooperating node
*D*	Destination node
*s*	Slice
$E_{S}^{s1}$	Energy consumption at S for sending data towards slice 1
$E_{S}^{s2}$	Energy consumption at S for sending data towards slice 2
$E_{S}^{s3}$	Energy consumption at S for sending data towards slice 3
$S_{e}^{1}$	Total sensors belonging to first layer
*n*	Total number of nodes
*N*	Set of n nodes
*N_U_*	Nodes deployed above to the center of the deployment field
*N_L_*	Nodes deployed below to the center of the deployment field
*D_e_*	Depth of the field
*d*	Depth between the transmitter and surface sink
$\hat{N}$	Set of neighbors
*N′*	Alive nodes
*η_c_*	Possible cooperating nodes set
*η_d_*	Possible destination nodes set
*B_c_*	Best cooperation set
*β*	Wireless link quality
*s_e_*	Sender node
*r_e_*	receiver node
*S_S,D_*	Signal sent from source and receive at destination
*S_S,C_*	Signal sent from source and receive at cooperating node
*S_C,D_*	Signal sent from cooperating node and receive at destination node
*α*	Channel responses
*n_S,C_*	Channel noises between source and cooperative relay
*n_S,D_*	Channel noises between source and destination
*x*	Original sent signal
$p_{z}^{D}$	Probability that z belongs to destination node
*M_AUV_*	Length of major axis
*m_AUV_*	Length of minor axis
*E*	Energy
*i*, *j*, *z*	Node’s ID
*h_i_*	Hop of i
$\rho_{h_{i}}$	Predecessors of hop i
*R_e_*	Residual energy
*P_e_*	Packet energy
*Dy*	Delay
*S_t_*	Sojourn time
*H*	Holding time
*P*	Packets generated
*C_cap_*	Channel capacity
*W_net_*	Network width
*T_xR_*	Transmission range
*T*	Transmission of packets

## References

[1] Akyildiz I.F., Pompili D., Melodia T. (2004). Challenges for efficient communication in underwater acoustic sensor networks. ACM Sigbed Rev..

[2] Shi Z.J., Fei Y. Exploring architectural challenges in scalable underwater wireless sensor networks. Proceedings of the Annual Boston Area Computer Architecture Workshop (BARC).

[3] Da P. (2014). Evaluation of Wi-Fi Underwater Networks in Freshwater. Ph.D. Thesis.

[4] Tan D.D., Le T.T., Kim D.S. Distributed cooperative transmission for underwater acoustic sensor networks. Proceedings of the Wireless Communications and Networking Conference Workshops (WCNCW).

[5] Silva A., Jesus S.M. A post-detection Maximum Ratio Combiner: Experimental assessment on high diversity underwater channels. Proceedings of the 2011 7th International Workshop on Systems, Signal Processing and their Applications.

[6] Lee J.W., Cheon J.Y., Cho H.S. A cooperative ARQ scheme in underwater acoustic sensor networks. Proceedings of the 2010 OCEANS IEEE-Sydney.

[7] Zhang Y., Chen Y., Zhou S., Xu X., Shen X., Wang H. (2015). Dynamic Node Cooperation in An Underwater Data Collection Network. IEEE Sens. J..

[8] Luo Y., Pu L., Peng Z., Zhou Z., Cui J.H., Zhang Z. Effective relay selection for underwater cooperative acoustic networks. Proceedings of the 2013 IEEE 10th International Conference on Mobile Ad-Hoc and Sensor Systems (MASS).

[9] Gao C., Liu Z., Cao B., Mu L. Relay selection scheme based on propagation delay for cooperative underwater acoustic network. Proceedings of the 2013 International Conference on Wireless Communications and Signal Processing (WCSP).

[10] Umar A., Javaid N., Ahmad A., Khan Z.A., Qasim U., Alrajeh N., Hayat A. (2015). DEADS: Depth and Energy Aware Dominating Set Based Algorithm for Cooperative Routing along with Sink Mobility in Underwater WSNs. Sensors.

[11] Wang C.F., Shih J.D., Pan B.H., Wu T.Y. (2014). A network lifetime enhancement method for sink relocation and its analysis in wireless sensor networks. Sens. J..

[12] Ahmad A., Javaid N., Khan Z.A., Qasim U., Alghamdi T.A. (2014). Routing Scheme to Maximize Lifetime and Throughput of Wireless Sensor Networks. Sens. J..

[13] Ahmed S., Javaid N., Khan F.A., Durrani M.Y., Ali A., Shaukat A., Sandhu M.M., Khan Z.A., Qasim U. (2015). Co-UWSN: Cooperative energy-efficient protocol for underwater WSNs. Int. J. Distrib. Sens. Netw..

[14] Cao J., Dou J., Dong S. (2015). Balance Transmission Mechanism in Underwater Acoustic Sensor Networks. Int. J. Distrib. Sens. Netw..

[15] Latif K., Javaid N., Saqib M.N., Khan Z.A., Qasim U., Mahmood B., Ilahi M. (2015). Energy Hole Minimization with Field Division for Energy Efficient Routing in WSNs. Int. J. Distrib. Sens. Netw..

[16] Javaid N., Jafri M.R., Ahmed S., Jamil M., Khan Z.A., Qasim U., Al-Saleh S.S. (2015). Delay-sensitive routing schemes for underwater acoustic sensor networks. Int. J. Distrib. Sens. Netw..

[17] Liao Y., Qi H., Li W. (2013). Load-balanced clustering algorithm with distributed self-organization for wireless sensor networks. Sens. J..

[18] Tarhani M., Kavian Y.S., Siavoshi S. (2014). SEECH: Scalable energy efficient clustering hierarchy protocol in wireless sensor networks. Sens. J..

[19] Thakkar A., Kotecha K. (2014). Cluster head election for energy and delay constraint applications of wireless sensor network. Sens. J..

[20] Gupta H.P., Rao S.V., Yadav A.K., Dutta T. (2015). Geographic Routing in Clustered Wireless Sensor Networks Among Obstacles. Sens. J..

[21] Lin H., Wang L., Kong R. (2015). Energy Efficient Clustering Protocol for Large-Scale Sensor Networks. Sens. J..

[22] Zhang S., Li D., Chen J. (2013). A link-state based adaptive feedback routing for underwater acoustic sensor networks. Sens. J..

[23] Javaid N., Jafri M.R., Khan Z.A., Qasim U., Alghamdi T.A., Ali M. (2014). Iamctd: Improved adaptive mobility of courier nodes in threshold-optimized dbr protocol for underwater wireless sensor networks. Int. J. Distrib. Sens. Netw..

[24] Yoon S., Azad A.K., Oh H., Kim S. (2012). AURP: An AUV-aided underwater routing protocol for underwater acoustic sensor networks. Sensors.

[25] Ahmed S., Javaid N., Ahmed A., Ahmed I., Durrani M.Y., Ali A., Haider S.B., Ilahi M. (2016). SPARCO: Stochastic Performance Analysis with Reliability and Cooperation for Underwater Wireless Sensor Networks. J. Sens..

[26] Javaid N., Jafri M.R., Khan Z.A., Alrajeh N., Imran M., Vasilakos A. (2015). Chain-based communication in cylindrical underwater wireless sensor networks. Sensors.

[27] Jorio A., El Fkihi S., Elbhiri B., Aboutajdine D. (2015). An Energy-Efficient Clustering Routing Algorithm Based on Geographic Position and Residual Energy for Wireless Sensor Network. J. Comput. Netw. Commun..

[28] Yu H., Yao N., Wang T., Li G., Gao Z., Tan G. (2016). WDFAD-DBR: Weighting depth and forwarding area division DBR routing protocol for UASNs. Ad Hoc Netw..

[29] Jing R., Kong L., Kong L. (2014). Boundary detection method for large-scale coverage holes in wireless sensor network based on minimum critical threshold constraint. J. Sens..

[30] Yu H., Yao N., Liu J. (2015). An adaptive routing protocol in underwater sparse acoustic sensor networks. Ad Hoc Netw..

[31] Baranidharan B., Santhi B. (2015). GAECH: Genetic Algorithm Based Energy Efficient Clustering Hierarchy in Wireless Sensor Networks. J. Sens..

[32] Jiang P., Liu J., Wu F. (2015). Node Non-Uniform Deployment Based on Clustering Algorithm for Underwater Sensor Networks. Sensors.

[33] Khan J.U., Cho H.S. (2015). A Distributed Data-Gathering Protocol Using AUV in Underwater Sensor Networks. Sensors.

[34] Hao K., Jin Z., Shen H., Wang Y. (2015). An Efficient and Reliable Geographic Routing Protocol Based on Partial Network Coding for Underwater Sensor Networks. Sensors.

[35] Uddin M.A. (2013). Link Expiration Time-Aware Routing Protocol for UWSNs. J. Sens..

[36] Shi L., Yao Z., Zhang B., Li C., Ma J. (2014). An efficient distributed routing protocol for wireless sensor networks with mobile sinks. Int. J. Commun. Syst..

[37] Javaid N., Ilyas N., Ahmad A., Alrajeh N., Qasim U., Khan Z.A., Liaqat T., Khan M.I. (2015). An Efficient Data-Gathering Routing Protocol for Underwater Wireless Sensor Networks. Sensors.

[38] Lucani D.E., Médard M., Stojanovic M. (2008). Underwater acoustic networks: Channel models and network coding based lower bound to transmission power for multicast. IEEE J. Sel. Areas Commun..

[39] Stojanovic M. (2007). On the relationship between capacity and distance in an underwater acoustic communication channel. ACM SIGMOBILE Mob. Comput. Commun. Rev..

[40] Brekhovskikh L.M., Lysanov I.P. (2003). Fundamentals of Ocean Acoustics.

[41] Domingo M.C. (2008). Overview of channel models for underwater wireless communication networks. Phys. Commun..

[42] http://www.arc.id.au/UWAcoustics.html.

[43] https://en.wikipedia.org/wiki/Relay_channel.

[44] http://www.mathwarehouse.com/ellipse/equation-of-ellipse.php.

